# *SLC26A4* gene copy number variations in Chinese patients with non-syndromic enlarged vestibular aqueduct

**DOI:** 10.1186/1479-5876-10-82

**Published:** 2012-05-02

**Authors:** Jiandong Zhao, Yongyi Yuan, Jing Chen, Shasha Huang, Guojian Wang, Dongyi Han, Pu Dai

**Affiliations:** 1 Department of Otolaryngology, PLA General Hospital, Beijing, People’s Republic of China; 2 Department of Otolaryngology, Hainan Branch of PLA General Hospital, Sanya, People’s Republic of China

**Keywords:** Enlarged vestibular aqueduct (EVA), MLPA, SLC26A4, Copy number variations (CNVs), Mutation

## Abstract

**Background:**

Many patients with enlarged vestibular aqueduct (EVA) have either only one allelic mutant of the *SLC26A4* gene or lack any detectable mutation. In this study, multiplex ligation-dependent probe amplification (MLPA) was used to screen for copy number variations (CNVs) of *SLC26A4* and to reveal the pathogenic mechanisms of non-syndromic EVA (NSEVA).

**Methods:**

Between January 2003 and March 2010, 923 Chinese patients (481 males, 442 females) with NSEVA were recruited. Among these, 68 patients (7.4%) were found to carry only one mutant allele of *SLC26A4* and 39 patients (4.2%) lacked any detectable mutation in *SLC26A4*; these 107 patients without double mutant alleles were assigned to the patient group. Possible copy number variations in *SLC26A4* were detected by SALSA MLPA.

**Results:**

Using GeneMapper, no significant difference was observed between the groups, as compared with the standard probe provided in the assay. The results of the capillary electrophoresis showed no significant difference between the patients and controls.

**Conclusion:**

Our results suggest that CNVs and the exon deletion in *SLC26A4* are not important factors in NSEVA. However, it would be premature to conclude that CNVs have no role in EVA. Genome-wide studies to explore CNVs within non-coding regions of the *SLC26A4* gene and neighboring regions are warranted, to elucidate their roles in NSEVA etiology.

## Introduction

Copy number variations (CNVs) or copy number polymorphisms are complex gains or losses of several to hundreds of kilobases of DNA as a result of deletions, insertions, duplications, and complex multi-site variants. CNVs are found throughout the human genome [1]. CNVs are much more frequent than chromosomal aberrations. They also encompass more nucleotide content per genome than single-nucleotide polymorphisms (SNPs) [2,3]. Therefore, CNVs may be more significantly correlated with phenotypic variations, and they may play vital roles in the evolution and development of species [4].

Multiplex ligation-dependent probe amplification (MLPA) was used to screen for CNVs of the *SLC26A4* gene in the present study. MLPA is a novel semi-quantitative method that was first developed by Schouten and colleagues in 2002 [5]. It allows the simultaneous processing of multiple sequences (up to 45) with various copy numbers. MLPA involves the hybridization of probes and target sequences, followed by ligation and PCR amplification. The amplified products are visualized by capillary electrophoresis, and the peak area and peak height of the amplified products are analyzed to reveal the relative copy number of the target sequence.

*SLC26A4*, which is also known as the Pendred syndrome (*PDS*) gene, is located on human chromosome 7q31. It contains 57175 bp and its mRNA is 4930 bp in length, encompassing 21 exons and containing an open reading frame (ORF) of 2343 bp. The ORF starts from exon 2 and continuously traverses the other 20 exons. *SLC26A4* encodes Pendrin, which has a molecular mass of 86 kDa and contains 780 amino acids [6]. Pendrin is a transmembrane protein that is mainly composed of hydrophobic amino acids. It is a member of the anion transporter family and contains an intracellular N-terminus, a C-terminus, and 12 transmembrane domains. Pendrin is expressed in the cells of the thyroid gland, kidney, and inner ear. Pendrin functions in the transportation of anions, including SO_4_^–^, HCO_3_^–^, CHOO^−^, C_2_O_2_^2–^, OH^–^, Cl^–^, I^–^, and C_6_H_12_O_6_ . Therefore, it plays a crucial role in maintaining the balance of ions [7]. In the inner ear, Pendrin is primarily expressed in the non-sensory structures, such as the external sulcus epithelial cells of the Corti organ and the endolymphatic duct and sac epithelial cells, and is involved in endolymphic fluid homeostasis. Pendrin, as a transporter of Cl^–^/HCO_3_^–^, regulates the ion balance of the endolymphic fluid [8]. Mutations in the *SLC26A4* gene may lead to autosomal-recessive deafness (DFNB4) and Pendred syndrome (non-syndromic enlarged vestibular aqueduct [NSEVA], inner ear malformation, sensorineural hearing loss, and goiter) [9].

*SLC26A4* gene mutations have the characteristic of allelic heterogeneity, i.e., deaf patients from different races may have a spectrum of mutations. In Northern Europe, T416P and IVS8 + 1 G > A are considered to be the most common mutations [10], while in France, the mutations are extremely diverse [11]. Among Asians, H723R is the most frequently observed mutation in Japanese and Korean populations [12,13], while 919-2A > G is the most common mutation in Chinese subjects [14,15]. One study has indicated that *SLC26A4* biallelic mutations (homozygote or complex heterozygote) cause NSEVA, while a person with a monoallelic mutation can only be a carrier [16]. However, many EVA patients have only one or no mutant allele of the *SLC26A4* gene. One explanation for the pathogenesis of these EVA cases is that the mutation is located within the promoter region or intronic cryptic splicing; these types of mutations are not readily detected by current screening approaches [17]. Another study has suggested the involvement of environmental factors or mutations in other genes [18]. However, these speculations have not been confirmed by relevant studies.

In the current study, we recruited patients with NSEVA who had no detectable *SLC26A4* mutation or only carried one mutant allele of *SLC26A4*. All the cases were diagnosed by the Molecular Diagnostics Center for Deafness, Institute of Otorhinolaryngology, PLA General Hospital. To date, no study of CNVs in *SLC26A4* has been conducted. In the present study, we used MLPA to screen patients for *SLC26A4* CNVs and to reveal the pathogenic mechanisms of NSEVA.

## Materials and Methods

### Recruitment of subjects

#### Patients with NSEVA

Between January 2003 and March 2010, 923 patients (481 males, 442 females) who were diagnosed as having NSEVA by computer tomography (CT) or MRI were recruited at the Molecular Diagnostics Center for Deafness, Institute of Otorhinolaryngology, PLA General Hospital. Among these patients, 262 (28.4%) carried *SLC26A4* homozygous mutations and 554 (60%) had complex heterozygous mutations. For the current study, 68 patients (7.4%) with one allele mutant and 39 patients (4.2%) without any detectable mutation were recruited. The average age of the 68 patients (32 males and 36 females) who carried one allele mutation in *SLC26A4* was 7.86 years (range, 3 months to 33 years; SD = 7.56). The average age of the 39 patients (23 males and 16 females) who lacked *SLC26A4* mutation was 9.65 years (range, 1–34 years; SD = 8.95). All the NSEVA cases were diagnosed by high-resolution CT or MRI. Vestibular aqueduct enlargement was diagnosed if the vestibular aqueduct was >1.5 mm in the middle portion of the descending limb in the axial plane, based on a CT scan of the temporal bone [19]. The MRI diagnostic criteria for vestibular enlargement included: diameter of the intra-osseous endolymphatic duct at its midpoint being >1.5 mm, an enlarged intra-osseous endolymphatic sac; and the T2-weighted signal intensity of the endolymphatic duct being designated as “high” [20].

#### “Healthy” controls

Controls were recruited from persons with a normal hearing examination and no family history of hereditary hearing loss. Genomic DNA samples were collected from these subjects as negative (wild-type) controls. In accordance with the MLPA experimental design criterion that the ratio of patients to controls should be 7:1, 16 controls (8 males and 8 females) were selected for the current study. The average age of the control subjects was 9.23 years (range, 2–36 years; SD = 8.76). No statistically significant difference in age was observed between the patients and controls.

All patients and controls signed informed consent forms, and this study obtained the approval of the People’s Liberation Army General Hospital Ethics Committee.

### DNA extraction, quantification, and quality assessment

DNA samples were extracted from the peripheral blood leukocytes using standard procedures. The extracted DNA was stored at −20°C until use. The DNA concentration and absorbance were measured in a spectrophotometer (Beckman Coulter DU800) at 280 nm and 260 nm. The ratio of absorbance readings at 260 nm and 280 nm (A260/A280) provides an estimate of the purity of the nucleic acid. All the extracted DNA samples had A260/A280 ratios of between 1.5 and 2.0.

### MLPA analysis

CNVs in the *SLC26A4* gene were detected using the SALSA MLPA KIT P280-A1 Pendred-SLC26A4 kit (MRC Holland, Amsterdam, The Netherlands). MLPA permits relative quantification of changes in the copy number of a specific genomic region. The P280 Pendred-SLC26A4 probe mix contains probes for each of the 21 exons of the *SLC26A4* gene. In addition, two mutation-specific probes were designed for the IVS8 + 1 G > A donor splice mutation and the T416P amino acid substitution. MLPA was performed in accordance with the manufacturer’s instructions (MRC Holland). Briefly, DNA samples were diluted with TE to 5 μl and heated at 98°C for 5 min. After cooling the samples to 25°C, 1.5 μl of probe mix (containing 1 fmol of each probe) and 1.5 μl of MLPA hybridization buffer were added, and the solution was denatured at 95°C for 1 min and hybridized at 60°C for 16 h. Hybridized probes were ligated at 54°C for 15 min after the addition of 32 μl of the ligation mixture. Following heat inactivation at 98°C for 5 min, 10 μl of the ligation reaction were mixed with 30 μl of PCR buffer, heated to 60°C, mixed with 10 μl of PCR mixture and amplified by PCR (35 cycles of 30 s at 95°C, 30 s at 60°C, and 1 min at 72°C, followed 1 cycle of 20 min at 72°C). The following PCR primers were used: forward, 5′-GGGTTCCCTAAGGGTTGGA-3′; and reverse, 5′-GTGCCAGCAAGATCCAATCTAGA-3′. The amplified products were analyzed in the ABI 3130 Avant capillary electrophoresis system. The genes and sequences recognized by the probes used in the present study can be found at http://www.mrc-holland.com.

## Results

### Characteristics of the study subjects

**Figure 1:**
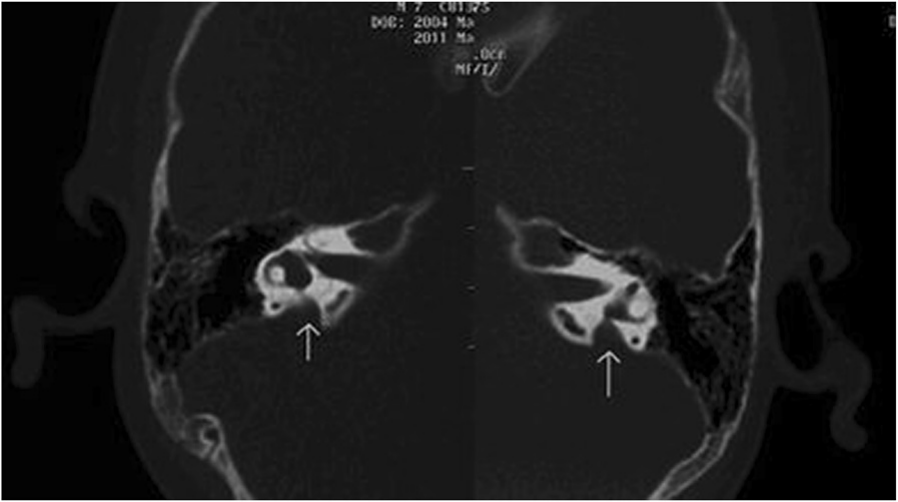
Image of the bilateral enlarged vestibular aqueducts.

The NSEVA group included 107 patients who either carry one allele mutation in the *SLC26A4* gene or lack a detectable mutation in *SLC26A4*. All cases were diagnosed and confirmed as NSEVA by CT and MRI scan (Figure 1). In total, 16 “healthy” controls were selected from subjects with a normal hearing examination and no family history of hereditary hearing loss. No significant differences were found for age or gender between the patients and controls (*p* < 0.05) (Table 1).

**Table 1:** Characteristics of the NSEVA cases and controls

	N	Age in years (SD)	Gender ratio (M/F)
NSEVA cases	107	8.73 (8.27)	1.1:1
Controls	16	9.23 (8.76)	1:1

### Comparisons of the experimental data for the patients and controls

**Figure 2:**
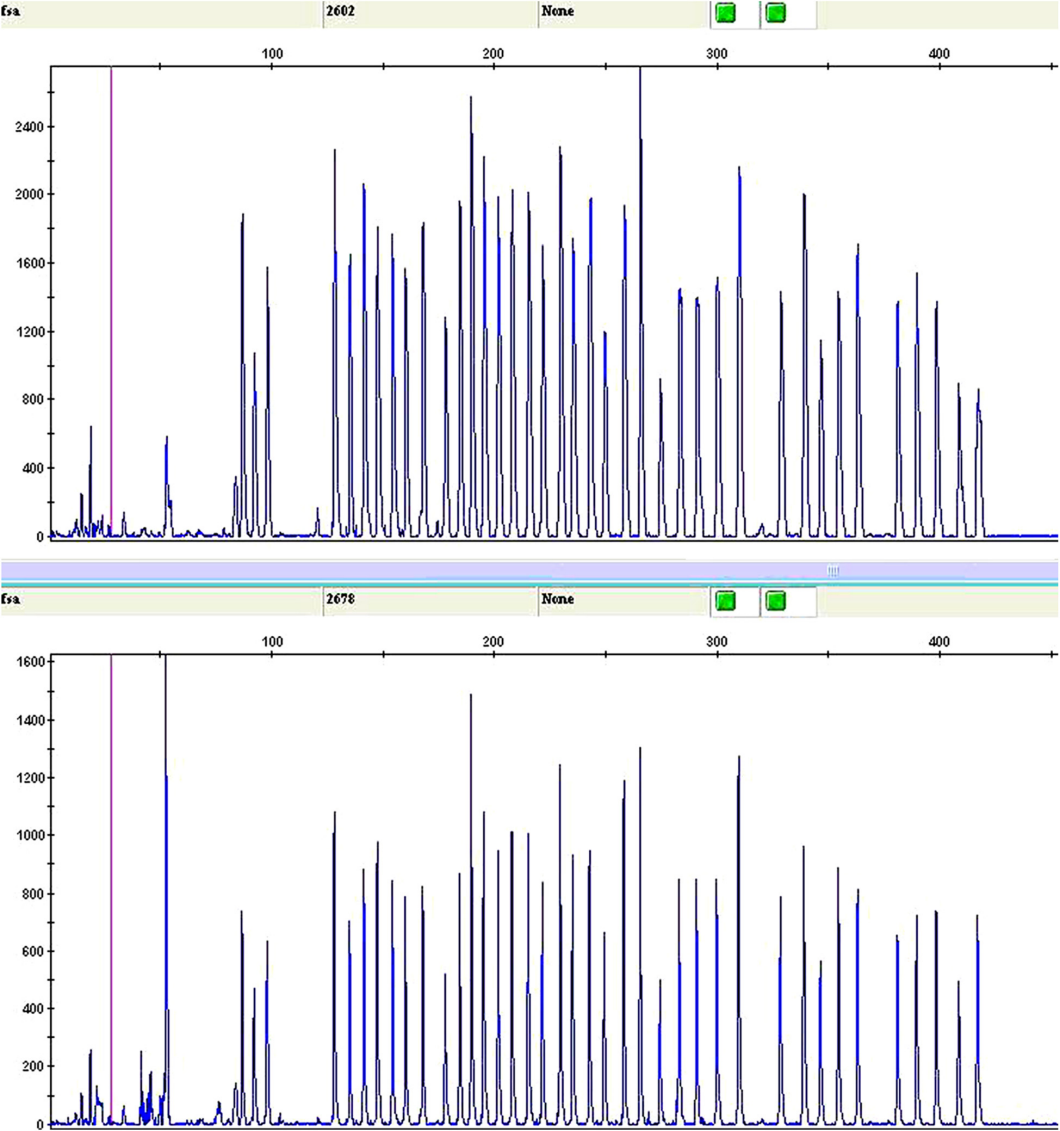
**GeneMapper analysis.** No significant difference was observed compared to the standard probe provided in the assay. The chart shows the results of capillary electrophoresis for one patient (upper panel) and one control subject (upper panel). There is no significant difference between the samples.

Experimental data were generated using the GeneMapper Software (ver. 3.7), and included six aspects: dye/sample peak; sample file name; size; height; area; and data-points. The preliminary data analyses did not reveal significant differences for capillary electrophoresis profile or for sample peak, size, height, area, and data-points. Some of the results are shown in Figure 2.

All the data were analyzed further using the Coffalyser MLPA Software, which generates a relative ratio from a comparison of the patients and controls. This ratio is regarded as ‘normal’ when it is within the range of 0.5–1.5. A ratio of <0.5 suggests a fragment deletion, while a ratio >1.5 indicates duplication. All the results for the 107 patients showed a normal ratio, which suggests that these patients lacked CNVs (deletions or duplications).

## Discussion

The difference in genome sequences between individuals is generally estimated to be less than 0.1%, and the differences are mainly manifested as SNPs. However, with the completion of the Human Genome Project, it is becoming clear that the genomic differences among healthy individuals are >0.1%, and include many variations [21]. The individual genomic variations may be quantitatively described as copy number gains (insertions and duplications), losses (deletions), transpositions (translocations and rearrangements), and inversions. Compared with SNPs, the frequency of CNVs is low, although the implicated sequences are much longer than SNPs, indicating that CNVs may have a greater impact on human diseases and health [22]. To date, more than 6,000 CNVs (http://projects.tcag.ca/variation) have been identified in the human genome. There have been reports of significant correlations between CNVs and deafness. For example, Knijnenburg et al. found a homozygous deletion on 15q15.3 in a CNV using comparative genomic hybridization (CGH) and MLPA [23]. This deletion of approximately 90 kb contains four genes, including the *STRC* gene linked to the *DFNB16* locus. The latter is known to be involved in autosomal recessive deafness [23]. Another study has reported that complex rearrangements of the 7q21.3 region are related to bilateral split-foot malformation and hearing loss [24]. MLPA, which is a semi-quantitative method with good sensitivity based on a simple quantitative PCR reaction, allows the simultaneous hybridization and ligation of probes. When this is followed by PCR amplification and capillary electrophoresis analysis, MLPA can detect multiple CNVs within DNA sequences [25,26]. This method has been widely used to study inherited diseases. In the current study, we applied MLPA to screen for CNVs in a deafness-related gene, *SLC26A4*.

The *SLC26A4* gene was first localized and cloned by Everett and colleagues from patients with Pendred syndrome [27]. The *SLC26A4* gene contains 21 exons and an ORF of 2343 bp. A clear demarcation was found between each intron and exon. The multiple exons observed in the gene indicate that it encodes a protein with a complex structure and function. The ORF of *SLC26A4* starts at exon 2 and continuously traverses the other 20 exons. The exons are 55 bp to 231 bp in length and encode the Pendrin protein, which contains 780 amino acids [6]. Pendrin is composed of hydrophobic amino acids and belongs to the anion transporter families. Its functions are associated with the transportation of anions, such as I^–^, Cl^–^, and HCO_3_^–^[28].

Pendrin is primarily expressed in the external sulcus epithelial cells of the Corti organ and the endolymphatic duct and sac in the inner ear. These non-sensory structures are correlated with the metabolism of endolymphic fluid. Pendrin, as a transporter of Cl^–^, may regulate the ion balance of the endolymphic fluid. It is well known that the homeostasis of the endolymphic fluid plays a pivotal role in the development of the inner ear. If homeostasis is disrupted, the osseous structures of the cochlea and vestibular aqueduct may be affected. Therefore, mutations in *SLC26A4* may lead to enlargement of the vestibular aqueduct [29].

Mutations in the *SLC26A4* gene are considered to be extremely heterogeneous [30]. The distributions of mutant *SLC26A4* alleles differ significantly among various ethnic populations [14,31,32]. Many different types of variations in *SLC26A4* have been found, including missense, nonsense, splice site, and frameshift mutations. However, there has been no report as to whether CNVs exist in the *SLC26A4* gene, which is the main topic of the current study.

We applied MLPA to screen for CNVs in the 21 exons of the *SLC26A4* gene in 107 patients with NSEVA who carry one or no mutant allele of *SLC26A4*. However, the results did not reveal any copy number gain or loss in the *SLC26A4* genes of these patients. There were several possible explanations for this observation. First, it is possible that no CNVs are present in the exons of *SLC26A4*, which would mean that CNVs are not the cause of the enlarged vestibular aqueduct. Second, CNVs may be present in *SLC26A4* but are located within the introns or lie upstream or downstream of the regulatory regions, i.e., not within the ORF. The MLPA assay is unable to detect these CNVs. Third, CNVs may exist in the genes adjacent to *SLC26A4* or in genes with regulatory functions for *SLC26A4*. These CNVs may alter the expression patterns or the functions of target genes, thereby indirectly affecting the *SLC26A4* gene, and ultimately causing enlargement of the vestibular aqueduct. Based on these results and interpretations, it is premature to conclude that CNV has no causative role in enlarged vestibular aqueduct. Genome-wide studies to explore CNVs within the non-coding regions of *SLC26A4* are warranted, to elucidate their roles in the etiology of NSEVA.

## Conclusion

Our results suggest that CNVs and the exon deletion in *SLC26A4* are not important factors in NSEVA. However, it would be premature to conclude that CNVs have no role in EVA. Genome-wide studies to explore CNVs within non-coding regions of the *SLC26A4* gene and neighboring regions are warranted in order to elucidate their roles in NSEVA etiology.

## Competing interest

The authors declare that they have no competing interests. This document has been checked by at least two professional editors, both native speakers of English. For a certificate, please see: http://www.textcheck.com/certificate/1rPIDr.

## Authors’ contributions

JD Zhao carried out the molecular genetic studies, participated in the sequence alignment and drafted the manuscript. YY Yuan carried out the immunoassays. J Chen participated in the sequence alignment. SS Huang and GJ Wang participated in the design of the study and performed the statistical analysis. DY Han and P Dai conceived of the study, and participated in its design and coordination and helped to draft the manuscript. All authors read and approved the final manuscript.
